# Implementing an early-life nutrition intervention through primary healthcare: staff perspectives

**DOI:** 10.1186/s12913-024-11582-z

**Published:** 2024-09-20

**Authors:** Natalie Garzon Osorio, Frøydis Nordgård Vik, Christine Helle, Elisabet Rudjord Hillesund, Nina Cecilie Øverby, Sissel H. Helland, Penelope Love, Mary Elizabeth Barker, Wim van Daele, Marianne Hope Abel, Harry Rutter, Tormod Bjørkkjær, Mekdes Kebede Gebremariam, Henrik Lian, Anine Christine Medin

**Affiliations:** 1 Department of Nutrition and Public Health, Faculty of Health and Sport Sciences, University of Agder, Kristiansand, Norway; 2 Institute for Physical Activity and Nutrition, School of Exercise and Nutrition Sciences, Deakin University, Geelong, VIC Australia; 3 School of Health Sciences, University of Southampton, Southampton, UK; 4 MRC Lifecourse Epidemiology Centre, University of Southampton, Southampton General Hospital, Southampton, UK; 5 Centre for Evaluation of Public Health Measures, Norwegian Institute of Public Health, Oslo, Norway; 6 Department of Social and Policy Sciences, University of Bath, Bath, UK; 7 Department of Community Medicine and Global Health, Institute of Health and Society, University of Oslo, Oslo, Norway

**Keywords:** First 1000 days, Nutrition, Digital intervention, E-learning resource, Healthcare, Primary care, Implementation, Implementation strategies, Qualitative methods

## Abstract

**Background:**

Nutrition interventions targeting early childhood can be cost-effective and may provide lifelong, intergenerational benefits. From October 2022 to April 2023 the *Nutrition Now* (NN) e-learning resource was implemented within Early Childhood Education and Care centres and the Maternal and Child Healthcare Centre (MCHC) in a southern Norwegian municipality. As part of the NN project, the present study aims to explore the MCHC staff’s experiences with implementing the NN resource, to gain insights into measures important to scale up digital early-life nutrition interventions.

**Methods:**

Three group interviews were conducted among public health nurses and midwives alongside one individual interview with the department leader of a MCHC in May 2023. An inductive thematic analysis, as described by Braun and Clarke, was conducted to generate the key themes and subthemes regarding the implementation process of NN within the MCHC.

**Results:**

Three main themes were generated: [1] *Important resource but not always utilized;* [2] *Parents are interested but had issues with access*; and [3] *Staff and stakeholder buy-in and commitment needed from the start.* Overall, the staff viewed the NN resource as a potential tool for promoting diet-related topics and believed it could support the guidance they were already providing parents. However, few staff members fully familiarized themselves with the resource. While staff perceived parents as positive when informed about NN, they believed issues such as access challenges, competing platforms, and time constraints reduced parental engagement. Lastly, staff suggested improvements for NN’s implementation, including enhanced training, better planning, assigning champions, and lowering the threshold for access.

**Conclusion:**

The findings of this study suggest that the real-world implementation of digital evidence-based health behaviour interventions is feasible but would be enhanced by employing strategies focusing on engagement and utilization.

**Trial registration:**

The main study is registered in the ISRCTN registry with ID ISRCTN10694967, 10.1186/ISRCTN10694967. (Registration date: 19-06-2022).

**Supplementary Information:**

The online version contains supplementary material available at 10.1186/s12913-024-11582-z.

## Background

The first 1000 days of life, the period from conception to a child’s second birthday, are an important window of opportunity to lay the foundations for long-term health [1, 2]. Recognizing the importance of the first 1000 days in shaping health trajectories, health behaviour interventions have increasingly been developed to target this critical life phase. Pérez-Muños et al. reviewed and analysed studies published between 2016 and 2021 and identified 51 interventions targeting the first 1000 days to prevent childhood obesity [3]. However, few of these interventions covered the whole 1000-day period, with most interventions (71%) ending either during the pregnancy or at the child’s birth [3].

This is unfortunate given that early childhood is a critical period in the life span, presenting risk exposures that are equally important to address to promote long-term health [4, 5]. Furthermore, while evidence-based early life interventions hold promise for public health improvement, their potential effectiveness relies on successful integration and sustained support within the settings and among the target groups they were designed for [6]. Additionally, it is uncertain to which extent such interventions have been implemented and sustained in real-world settings. It is well recognized that there is little guidance available providing practical and systematic approaches for implementing early childhood health behaviour interventions in real-world settings, representing a challenge for the transfer of research into practice [7]. The field of implementation science, aiming to enhance the successful integration of evidence-based interventions into real-world practices, addresses this challenge [8]. It seeks to do so by identifying the enablers and barriers to dissemination, adoption, implementation, and integration of interventions across settings, and developing strategies to support these processes [9, 10]. Understanding the processes needed to support the adoption, implementation, and integration of health behaviour interventions targeting the first 1000 days of life, in addition to evaluating their effectiveness, is therefore important for real-world delivery at scale.

Building on the recognized significance of implementing interventions targeting the first 1000 days, we designed the *Nutrition Now* (NN) project [11], introducing a new e-learning resource to improve dietary habits and related behaviours in early life. Briefly summarized, the NN resource is an adaption of four interventions with proven efficacy [12–16], that target diet during pregnancy, parental feeding practices in infancy and toddlerhood, and diet quality and meal environments in Early Childhood Education and Care (ECEC) centres.

The adapted resource is an online website available in Norwegian, English, and Arabic that presents key aspects of dietary transitions from pregnancy to a child’s second birthday. These topics are presented through videos, graphics, images, and short texts, addressing: (1) the importance of diet early in life; (2) promotion of breastfeeding; (3) the parental role in food provision and shaping a child’s diet; (4) responsive feeding; (5) shared meals; (6) knowledge and experience of food preparation; (7) sensory play with vegetables; and (8) collaboration between ECEC centres and parents. Parents were informed about the resource through the Maternal and Child Healthcare centre (MCHC), the participating ECEC centres, and social media. To access the resource parents had to enrol in the NN study by submitting a baseline questionnaire that provided sociodemographic information. Once enrolled, they could freely use the resource. Additionally, they received regular newsletters by email featuring content from the resource tailored to their child’s age.

The content of the NN resource was primarily designed to target parents and staff working in ECEC, but it could also be used by MCHC staff to support their guidance on food and nutrition-related topics. MCHC staff gained access to the resource using direct links or QR codes provided to them in an informational brochure.

The NN project has two phases, as described more in detail in the study protocol [11]. Phase 1 involves implementation and effectiveness evaluation in a municipality setting which will inform the subsequent scale-up to county settings in phase 2. It is important to gain an understanding of MCHC staff’s perspectives on having NN available within their setting during this first phase of the study to inform the phase 2 scale-up of NN at the county level. Evaluations from the Early Education and Care centres have been conducted in a separate study (unpublished observations, Lian H. et al.). The main objective of this qualitative study within the NN project was therefore to explore the implementation of the NN resource within an MCHC setting, focusing on MCHC staff’s perspectives on utilization, promotion to parents and implementation strategies. By including key staff members working within the MCHC setting, who we intend to target when scaling up the intervention, we aimed to answer the following research question: *Through consideration of MCHC staff’s input*,* which efforts could be important to prioritize to help scale up digital early-life nutrition interventions such as NN within MCHC settings?*

## Methods

We conducted a qualitative study using group interviews with public health nurses and midwives and one individual interview with the MCHC department leader to explore their experiences with implementing the NN resource. Qualitative methods were applied in the present study as they are suitable for providing a deeper understanding of experiences, phenomena, and context, and are appropriate when detailed insights into research questions are needed [17, 18]. Additionally, such methods are an integral part of implementation research, contributing to providing important answers to *how* and *why* efforts to implement interventions may succeed or fail [19]. Group interviews allow the capturing of diverse perspectives and experiences and enable more in-depth and nuanced exploration of the topics under discussion. It was therefore considered an appropriate method to explore public health nurses’ and midwives’ perspectives of the NN implementation effort. The individual interview was conducted to include the views of the MCHCs department leader. The department leader held a pivotal role in establishing the NN project within the MCHC and was involved during the active implementation phase, but not to the same extent as the public health nurses and midwives. Nevertheless, the department leader was included to gain insights and representation across staff levels within the MCHC. The Consolidated Criteria for Reporting Qualitative Research checklist guided the reporting of this study [20].

### Study setting

This study is part of the first phase of the NN project carried out in a municipality in the southern part of Norway [11]. In 2023 the municipality had 46,213 residents, of which 2064 were children aged 0–4 years, and 411 were newly registered pregnant women who had attended prenatal care. Within the municipality, there is one sole MCHC in charge of providing public health services to the population, all free of charge. As of October 2022, the medical staff of this service consisted of 14 public health nurses and five midwives, with one department leader managing all units. In the NN project, the MCHC’s midwifery service and the healthcare service for children aged 0–5 years were designated to implement and disseminate the NN resource to prospective parents or parents with children aged 0–2 years. The MCHC was considered an appropriate setting to inform parents about the NN resource as its services are widely used among the Norwegian population. In Norway, all pregnant women are entitled to nine antenatal consultations provided by the MCHC’s maternity service or by their general practitioner [21]. Through the MCHC’s infant healthcare program, parents are then offered regular consultations as part of a standardized schedule, consisting of 14 consultations [22]. These consultations begin 7–10 days after birth and continue regularly until the child reaches four years of age [23]. Approximately 98% of all Norwegian children are in contact with their municipal MCHC within their first year of life [24], making MCHC staff uniquely positioned to deliver health promotion interventions reaching all population groups in society.

The active implementation phase of NN lasted for 6 months, from October 2022 to April 2023. For MCHC staff this entailed having the NN resource available at the MCHC and providing pregnant people and parents with information about and access to the resource. To enhance the likelihood of resource utilization and promote uptake in the target setting and among the target group, twelve implementation strategies from the Expert Recommendations for Implementing Change (ERIC) [25] were selected. The strategies are described in detail in the NN Phase 1 protocol [11], but an overview is provided here in Table 1. Nine out of twelve implementation strategies were used in the exploration and preparation phases before the active implementation started in October.

**Table 1:** Overview of implementation strategies from the Expert Recommendations for Implementing Change (ERIC) taxonomy targeting NN implementation through the MCHC

Implementation phase	Implementation strategy	Action target (affected by strategy)
Exploration and preparation	#4 Assess for readiness and identify facilitators and barriers	MCHC staff
	#18 Conduct local needs assessment	
	#6 Build a coalition	
	#17 Conduct local consensus discussions	
	#38 Inform local opinion leaders	
	#15 Conduct educational meetings	
	#31 Distribute educational materials	
	#29 Develop educational materials	MCHC staff, pregnant women, parents
	#51 Promote adaptability	Pregnant women and parents
Active implementation	#63 Tailor strategies	Pregnant women and parents
	#58 Remind clinicians (including end users of the NN resource)	Pregnant women and parents
	#56 Purposefully reexamine the implementation	MCHC staff

Implementation strategies #4, #18, and #17, which involved interviews, focus groups, and workshops with MCHC staff, were employed to identify implementation facilitators and barriers and to tailor resource content (strategy #51). Implementation strategies #6, #38 and #15 sought to facilitate collaboration between staff and improve their understanding of the NN resource and the project’s fundamental principles through educational meetings. Implementation strategies #29, #63 and #31 included developing and distributing educational materials for parents and staff, including brochures for staff detailing project objectives and encouraging familiarity with the resource. Informational cards with QR codes linking to the NN resource, posters, and stickers with the NN logo were provided to the MCHC for distribution to parents. During active implementation, parents received tailored reminder emails with updated resource content (strategy #58). Lastly, monthly telephone interviews were conducted with coordinators of midwifery and healthcare services to monitor progress and offer support (strategy #56).

### Participant recruitment and data collection

Data collection took place in May 2023. All staff working at the MCHC at the time of data collection were involved in implementing the NN project and delivering information about the resource to parents. All these staff members were eligible to participate in the study. To recruit participants, initial contact was made by NGO, a PhD student, who emailed the public health nurse coordinator, the midwife coordinator, and the department leader to explain the study’s aim and schedule the group interviews and the individual interview. The coordinators were responsible for conveying information and recruiting participants for the group interviews. Since all eligible participants were working within the only MCHC in the municipality, group size for interviews was determined by the availability of the staff. As presented in Table 2, three group interviews were conducted, with each including five public health nurses, two public health nurses, and four midwives, respectively. Additionally, one individual interview was conducted with the department leader to achieve representation across different levels of staff.

**Table 2:** Overview of interviews conducted with Maternal and Child Healthcare staff

Interview type	*n*	Duration
Group interview with public health nurses	5	119 min
Group interview with public health nurses	2	53 min
Group interview with midwives	4	84 min
Individual interview with department leader	1	30 min

The group interviews and the individual interview were facilitated by NGO and a post-doctoral fellow (CH) experienced in qualitative research. As part of ERIC strategy #56, NGO conducted monthly short interviews with one public health nurse and one midwife during the active implementation phase. Additionally, CH was involved in interviews, focus groups, workshops and educational meetings with some of the MCHC staff during the exploration and preparation phase of the NN project. As a result, the facilitators were familiar with some of the participants prior to data collection.

The group interviews took place at the MCHC during work hours, lasted between 50 and 120 min, and were facilitated by NGO and CH. Two dictaphones were used for audio recording the group interviews. The individual interview was conducted virtually and recorded via Zoom, lasted approximately 30 min, and was facilitated by NGO. Interview facilitators used semi-structured interview guides, including both close-ended and open-ended questions (Additional file 1). These aimed to capture MCHC staff’s experiences with the implementation of the NN resource, the perceived relevance of implementation strategies, and reflections regarding sustaining the implementation, both among staff and parental users. Written informed consent was obtained from all participants before conducting group discussions and interviews, and only participants and facilitators were present during all interviews.

### Analysis

The audio files were uploaded to the project’s secure server, the Service for Sensitive Data (TSD, in Norwegian) [26], and auto-transcribed using the Whisper software [27]. The transcripts were then uploaded to NVivo 12 [28] for cleaning, which involved checking the transcripts for misspellings, removing all identifiable identifications, and assigning new personal identifiers to the participants. Due to considerations of participation burden, transcripts were not returned to participants for member checking.

The transcripts were analysed using inductive thematic analysis. NGO, CH, ACM and FNV followed the six-phase process of reflexive thematic analysis, as described by Braun and Clarke [29]. This approach was chosen as it provides a flexible analytical method appropriate for identifying meaning patterns across different types of data [30]. In the present study, the first phase of analyses involved data familiarization in which NGO, CH, ACM and FNV read through the transcripts individually. First impressions were discussed in group meetings. The second phase involved initial inductive coding, where transcripts were coded line by line by NGO, summarizing the main ideas or concepts. In the third phase, NGO collated codes sharing core ideas or concepts to generate initial themes. In the fourth, fifth and sixth phases of the thematic analysis, the initial themes were reviewed and refined. In these phases, NGO, CH, ACM and FNV met regularly in consensus meetings to discuss whether the themes and subthemes captured the data accurately. When reaching an agreement on the candidate themes and their congruence with the coded dataset, the final thematic themes were defined and named.

In the present study, all quotes presented have been translated from Norwegian to English by a bilingual author (NGO) using publicly available machine translation services and the author’s discretion. To maintain participant anonymity all 12 MCHC staff, including the department leader, were designated as “Speaker” with an assigned number from 1 to 12 (e.g., Speaker *5*).

Furthermore, the present qualitative study is situated within the interpretive paradigm, underpinned by a relativist ontology and subjective epistemic position, in which reality is shaped by social interactions and personal experiences, and meaning is created through the interplay between the researcher and participant, emphasizing that knowledge and reality are co-constructed and dependent on context [31–33].

## Results

The participants in this study were all women and exhibited a range of experience, with years worked at the MCHC varying between 2 and 33 years, and years qualified as a nurse or midwife ranging from 16 to 40 years.

Inductive coding procedures generated three overarching themes representing staff’s perspectives on the implementation of NN through the MCHC (Table 3). An overview of the coding tree with representative quotes is provided in Additional file 2. In the following sections, themes and sub-themes are summarized with illustrative quotes, taken either from the individual interview or group interviews.

**Table 3:** Main themes and sub-themes generated through thematic analysis

Main themes	Theme 1: Important resource but not always utilized	Theme 2: Parents are interested but had issues with access	Theme 3: Staff and stakeholder buy-in and commitment needed from the start
**Sub-themes**	• The resource aligns with the existing practices and priorities • Perceived as a reliable supporting tool • Lack of personal familiarity with the resource	• Parents seemed interested but many did not log in • Accessing the resource presented challenges • Parents are subject to other competing factors	• Informational material was well received • Multilevel buy-in processes are needed • Improvements to implementation o Improvements in the MCHCs o Improvements for parents

## Theme 1: Important resource but not always utilized

MCHC staff had a positive attitude towards NN, believed it was suitable for integration into their routines, and saw potential in being able to offer this as a tool to parents to help address diet-related topics of their concern. Nevertheless, there seemed to be significant room for improvement in terms of engaging staff to use the resource.

### The resource aligns with the existing practices and priorities

Across all discussions, it quickly became clear that nutrition is a topic deemed important by MCHC staff and frequently inquired about by parents. However, although nutrition-related topics were addressed in nearly every consultation, some expressed that these matters deserved even greater emphasis.

*We talk about diet a lot*,* or… I wished we talked more about it. But it’s a topic we should cover in every consultation*,* so to speak*,* but you don’t always get as much time as you’d like.* (Speaker 6)

Furthermore, the NN resource aligns with the national dietary guidelines, which also guide MCHC staff, resulting in a natural overlap between the resource’s content and the staff’s thematic priorities in discussions with parents. For instance, one staff member described that it had been nice presenting the NN resource as it was supportive of the dietary advice they were already providing.

### Perceived as a reliable supporting tool

Overall, MCHC staff had a positive attitude towards the NN resource. They perceived the resource as a reliable source of quality assured nutrition information and seemed to appreciate that it was free of advertising. Some also viewed it as positive that they could provide nutritional information in multiple languages beyond what they already communicate verbally and stated that they found it easy to inform parents about the resource. Furthermore, staff viewed the resource to be supporting for them in that it helped reinforce their emphasis on the importance of nutrition to parents. The NN resource was thus perceived as a relevant tool for MCHC staff to address nutrition-related topics.

*Nutrition Now is underpinning what we otherwise talk about […] so I feel it has been a tool for us to discuss food and lifestyle in a good way.* (Speaker 8)

### Lack of personal familiarity with the resource

While MCHC staff expressed a positive attitude toward the NN resource, few had accessed the resource or utilized it during consultations. Some stated that they found the login procedure to be cumbersome while others stated that they simply had forgotten to log into the resource themselves. Some of those who stated that they had not logged into the resource expressed that in hindsight they felt they should have done so to become more familiar with the resource’s content, and it appeared that a lack of familiarity influenced how they engaged with parents about it.

*When I was informing*,* I recognized that I hadn’t logged in myself. I should have done that. […] When they [parents] then have asked questions*,* I haven’t really been able to answer.* (Speaker 5)

On the other hand, one participant expressed that although they were not familiar with all the contents of the resource, they were well familiarized with the NN project overall. Additionally, when reflecting on why they had not logged into the resource, some believed that the nutritional expertise of the staff was good, perhaps influencing their perceived need to explore the resource more thoroughly.

*We have a group of very experienced nurses who feel we know the basics of nutrition counselling. So*,* we may not have had a great need for it.* (Speaker 1)

## Theme 2: Parents are interested but had issues with access

Staff perceived parents to be positive and interested when informed about the resource but were unsure whether parents engaged with the resource. Some staff members received feedback from parents about difficulties in accessing the resource. They believed these challenges, along with other factors such as the availability of competing nutritional platforms and time constraints, influenced parental engagement.

### Parents seemed interested but many did not log in

From the MCHC staff’s perspective parents were mostly positive when informed about NN and the staff perceived that the information was well received. Some of the MCHC staff perceived the most engaged parents to be those who find nutrition-related topics intriguing or have a need to discuss them in the first place. MCHC staff also expressed that it seemed to have been especially well received among parents who had children in the ECEC centres, and experienced that these parents were more engaged than others.

*I’ve received many of the parents who have children in the ECEC centres again. They are very positive. They say “Yes*,* we are familiar with that [NN]*,* because we have that in the ECEC centre*,* and it’s very good”. (Speaker 9)*

Although MCHC staff perceived parents to be mostly positive and interested when informed about the NN resource, a few staff members noted that certain parents may have had elevated expectations as to what the resource encompassed. Additionally, staff noted that parents are often provided with large amounts of information during one consultation, and there were uncertainties among some regarding how parents utilized the information they received afterwards. For instance, some staff members expressed that they had to provide parents with several reminders to use the resource, but that despite this, some parents still forgot to make use of it. During discussions with staff as to why parents had utilized the resource or not, some of the staff stated that subjects such as nutrition and diet should be addressed and discussed individually with each parent and suggested that parents might prefer engaging in face-to-face conversations about nutrition-related topics rather than being directed to a resource.

*I think it is important that we don’t just hand out the card [with access to the resource] and then we’ve addressed the topic of diet. We need to address it individually and talk about it. (Speaker 6)*

### Accessing the resource presented challenges

Almost all MCHC staff expressed that parents had encountered difficulties accessing the resource. Parents needed to provide baseline information in the form of online questionnaires before gaining access to the resource, and from the MCHC staff’s perceptions, this was pointed out as being an overly cumbersome process for parents. A few parents had reported to the MCHC staff that the questionnaires were too long and invasive. Some of the staff also mentioned that, in the start-up phase, some parents reported not gaining access to the resource despite responding to the questionnaires. The issues related to parental access ultimately affected the staff as well, with some expressing that they found it demotivating. One staff member emphasized that promoting the resource to parents would have felt easier if it was not associated with a questionnaire-based login process.

*I’ve been feeling that a bit*,* that I offer it [the resource]*,* but then I know that you [the research group] will collect something from them […]. I wish they could just go in and obtain information [from the resource] without having to do so much. (Speaker 4)*

### Parents are subject to other competing factors

In discussions, reflecting on their experiences with promoting NN to parents, staff members shared thoughts regarding factors perceived to affect parental engagement. Competing platforms was named as one possible hindrance to engagement. Staff noted that parents sought dietary information from a variety of sources, including Norwegian government-supported websites (e.g. Matportalen and Ammehjelpen), as well as non-government-supported platforms like the commercial app NØRS, and social media platforms such as TikTok and Instagram. One staff member acknowledged that convincing parents to opt for NN rather than these alternative commercial platforms could pose a challenge.

*[…] these dietary guidelines from Matportalen [website]*,* parents use that a lot*,* and NØRS [app]*,* a lot of people use that too. Clearly*,* there are many other apps and sites to access. Which are competing with Nutrition Now in a way. (Speaker 10)*

A couple of the MCHC staff also brought up time constraints among parents as a consideration. They acknowledged that parents with young children are navigating a hectic phase of life and may require faster ways to acquire information (e.g., apps) or simply more time in their daily routines to sit down and explore a resource.

## Theme 3: Staff and stakeholder buy-in and commitment needed from the start

Staff were generally positive about the way the intervention was being implemented but offered suggestions as to how the implementation of NN could be enhanced to foster engagement, both among staff and parents.

### Informational material was well received

Almost all MCHC staff expressed that they enjoyed having small informational cards available for distribution. One staff member expressed that offering parents something tangible was valuable, as parents are inundated with information during consultations. Furthermore, it was highlighted by several individuals that cards, stickers, and posters served as reminders for staff to talk about NN, and some believed they functioned as a reminder for parents as well. One of the staff members further pointed out that she was pleased that both the resource and the cards were available in multiple languages, facilitating the provision of more in-depth dietary-related information in Arabic and English.

*It has been easier to talk about diet after getting those [cards] […]. It reminded me to hand them out and […] it created like a little pocket there to talk about it. (Speaker 7)*

### Multilevel buy-in processes are needed

Prior to the implementation start-up, some of the MCHC staff voluntarily participated in workshops, and most MCHC staff attended a preparatory meeting during working hours, where researchers informed them about the NN resource. It was clear across the discussions that the staff considered these meetings important, helping them create a backdrop for introducing the resource to parents.

When asked about buy-in processes needed for future scale-up of NN implementation, involving all MCHCs in a county setting, employees expressed the importance of involving high-level leaders to ensure sufficient buy-in within the different municipalities. Several also emphasized the importance of involving the leaders at the respective MCHCs but stressed that the most crucial aspect was to engage all staff from an early stage to avoid top-down pressure and feeling forced to make use of the NN resource. Furthermore, some of the staff members expressed that to engage new MCHCs in joining the project, it would be necessary to inform them about NN in person through dialogue, either through digital or physical meetings.

*People need to have a sense of ownership. That’s the most important thing. If you’re going to implement it*,* the employees have to think that “Yes*,* this is us and this will help us in our work”. And that means having to meet up*,* or I think we need to talk to each other*,* to get that feeling*. (Speaker 8)

### Improvements to implementation

During the discussions, MCHC staff provided several suggestions for future implementation efforts, both within the MCHC and to foster parental engagement.

#### *Improvement in the MCHCs*

Suggested measures at the MCHC included allocating dedicated time during specific consultations to introduce NN to parents and establish this as an independent procedure. Examples included the consultations at four months or six months, where transitions to solid foods is a commonly discussed topic. On the other hand, such an initiative would require a good understanding of what the resource can offer and several of the staff members mentioned that familiarizing oneself with the content of a resource is a time-consuming process. However, they recognized that engaging in workshops to familiarize themselves with the resource could be beneficial. Such an approach could empower the staff not only to explore optimal ways of integrating NN at the MCHC but also to build confidence in utilizing a new innovation.

*It’s about being confident and knowing the tool you’re using. So*,* if I had become familiar with the NN site*,* I could easily be like “here you have this and…”. But that requires an effort from us then.* (Speaker 7)

Furthermore, several staff members pointed out that it was important for the management to follow them up going forward, reminding them to use the NN resource. Others also pointed out that it might be wise to have a designated person with overall responsibility for NN, as this is something they already do with other initiatives.

*The management must remind people that this exists. That there are brochures*,* and that posters are put up*,* and that materials are easily accessible to everyone. That it becomes something we talk about.* (Speaker 11)

#### *Improvement for parents*

The MCHC staff also had many suggestions for measures that could be initiated to engage parents in utilizing the resource to a greater extent. One of these measures was to tailor the timing for the introduction of NN to parents. For instance, several staff members expressed that it might be more beneficial to introduce NN when parents themselves express that they need more guidance. Additionally, they said the perceived relevance for parents might diminish if the resource is introduced either too early or too late in relation to their child’s development. Furthermore, as a measure to simplify the accessibility of the resource, several participants expressed that it could be beneficial to integrate NN into existing platforms already used by parents and frequently referred to by staff, thereby consolidating all relevant resources in one place. Lastly, staff members emphasized the importance of keeping the resource up to date, both in terms of design and content, to ensure relevancy.

*You realise that the years go by*,* and things quickly become outdated. It may be relevant to look at the resource to see if we need to update it a little. So that it always seems new and updated for parents who are going to use it. I think that’s very important […] to make it interesting for the population.* (Speaker 12)

## Discussion

The objective of this study was to investigate MCHC staff’s perspectives on the implementation of the NN resource, focusing on their utilization, promotion to parents and implementation strategies.

Our findings that MCHC staff were positive towards NN and perceived it to be a supportive tool, are similar to those of other studies exploring healthcare professionals’ experiences and perceptions of digital resources [34, 35]. In one such study, Alexandrou et al. (2023) explored Swedish primary healthcare nurses’ user experiences with the MINISTOP 2.0 app, developed to promote healthier eating and physical activity in toddlers [34]. They found that healthcare nurses believed it could be beneficial to have an evidence-based digital support tool available as a complement to their daily practice and the conversations they already offered parents [34]. Similar to the findings from the present study, they also found that nurses viewed the content of the app to be in line with the messages they already strive to convey [34]. Another Swedish study, exploring midwives’ and nurses’ perceptions of a digital support tool aiming to prepare parents for childbirth (Childbirth Journey), also emphasised how digital tools were primarily viewed by staff as an extension of the professional support they already provide parents [35]. Ensuring that digital resources are perceived to be useful for the healthcare setting in which they will be applied seems to be important, and perceived usefulness has been described as one of the main facilitators for the utilization and adoption of digital health technologies by health professionals [36]. However, it is important to acknowledge that the healthcare professionals across the MINISTOP 2.0, Childbirth Journey and NN interventions viewed digital tools to be complementing the services they already offer parents. This might influence how healthcare professionals engage with such resources and may explain why the MCHC staff in the present study, despite having positive inclinations towards it, did not particularly engage with the resource. Nevertheless, given that recent reviews reveal that parent-focused digital nutrition and health interventions can positively change children’s dietary intake and behaviours [37, 38], it is encouraging that MCHC staff in the present study generally seem supportive of incorporating such digital resources within their setting.

An important finding from the present study is how, despite having the NN resource readily available, few MCHC staff had visited the resource and become acquainted with its content. This lack of familiarity may have affected their ability to endorse and promote the resource to parents, potentially limiting parental engagement with NN. This challenge highlights the importance of ensuring stakeholders are well-acquainted with the interventions being implemented, to be comfortable and confident in their use and dissemination.

A possible explanation for the lack of familiarity could be attributed to staff members expressing confidence in their ability to provide parents with nutritional counselling, potentially diminishing their perceived need to acquaint themselves with the content of the NN resource. Moreover, several implementation strategies were employed before implementation start-up, which entailed the involvement of MCHC staff to help inform the development of the NN resource, create a collaborative environment, and increase their knowledge about the NN resource and project overall. Staff were also provided with a brochure in which they were encouraged to familiarize themselves with NN. It is possible that these activities led staff to feel sufficiently informed about the resource, reducing their inclination to engage with it more deeply.

While studies have shown that involvement in development and implementation helps facilitate the adoption of digital health technologies among healthcare professionals [36], our findings suggest that providing educational meetings and informational materials alone was insufficient to fully engage MCHC staff in utilizing the resource in their daily work. Furthermore, it is important to note that some of the staff acknowledged that if they had known the resource better it would have been easier to disseminate it to parents and include it as part of the guidance they were delivering.

Providing MCHC staff with clear guidance to illustrate how the resource can complement the existing practice, and additional training sessions during implementation to ensure exposure and utilization among staff, could be explored in future scale-up efforts. Previous studies have described training and educational activities to be important facilitators for digital health technology adoption [36], and for sustaining implementation efforts [39, 40]. Moreover, MCHC staff in the present study viewed these educational and preparatory meetings to be valuable. This might indicate that such meetings are important for implementation buy-in, but that there needs to be a larger focus on the practical applicability of the resource within the MCHC setting, in addition to providing general information about the innovation, to help facilitate engagement among staff. The staff themselves suggested that as a group, they should spend more time becoming acquainted with the resource, which could facilitate their decision-making regarding when and under what circumstances it would be appropriate for them to utilize the resource. A strategy from the ERIC taxonomy that could support such a process is to develop an implementation blueprint, in which employees create their own plan for integrating the resource into their practice. Such a plan may involve employees reflecting on what they would aim to achieve by having the resource available at their MCHC, who should be involved to ensure the resource is utilized, and when and how they make use of it. Developing implementation blueprints has been described as a collaborative process that can engage members who will be responsible for implementing innovations, with the potential to build buy-in, enhance fit, and improve the likelihood of successful implementation [41–43].

Such a strategy could potentially also serve as a solution to staff’s concerns regarding parental engagement. The staff mentioned challenges in assessing whether parents made use of the resource, questioned whether parents had the availability to engage with it, and believed it competed with other platforms. Developing blueprints could help staff become well-acquainted with the resource, which in turn could help them advocate for its use and recommend modules from the resource that are more tailored to parents’ needs. Additionally, the staff proposed incorporating NN during consultations that frequently involve discussions about food and meals. The development of blueprints may assist in pinpointing the most relevant aspects of the resource to present to parents during these specific consultations. Consequently, adopting a more targeted approach in introducing NN to parents could heighten parents’ perceived relevance of the resource. A scoping review of child health promotion apps for parents supports this assertion, revealing that apps are predominantly used when they can address parents’ needs and questions regarding their child’s health when immediate answers are sought [44]. Similarly, focusing the message by tailoring the contents of an intervention to the parents’ needs has been identified to be one of the most important facilitators to engagement in parenting programs [45].

Additionally, staff expressed that NN has to compete with many other similar platforms, possibly challenging its relevance and visibility among parents. For an innovation to be effectively implemented, it must be perceived as advantageous compared to alternative solutions [46]. Staff concerns regarding the perceived relevance of the resource among parents might indicate the anticipated benefits of utilizing the resource have been ineffectively communicated, both to MCHC staff in educational meetings and to parents through the website. Another potential factor could be associated with the long-term nature of these benefits, making it difficult for parents to perceive immediate value. This is unfortunate, as studies have shown that observability - the degree to which results of an intervention are visible to the recipient - is one of the most influential attributes for innovation uptake [47]. Considering staff suggestions, increasing the relevance and visibility of the NN resource could be achieved by integrating it into platforms that are already widely used by parents. This approach could address the potential barrier of limited program awareness, which has been suggested to hinder parental engagement in early childhood obesity prevention programs [48]. Additionally, promoting programs through multiple channels with information clearly conveying the content and benefits of programs have been suggested to facilitate parents’ engagement with programs [48, 49]. In our study, promoting the resource across different settings in which young children and their parents interact seem to have positively impacted participants’ impressions and familiarity with the resource. Collaboration to promote innovations across settings, and ensuring integration into platforms already familiar to parents are thus two measures that may be relevant to sustain and incorporate for future scale up efforts.

Lastly, another proposed barrier to parental engagement was the extensive questionnaire-based login process required for effectiveness measures during the project’s first phase. Combining implementation and effectiveness research may have been a hindrance to the participation and subsequent engagement. As the NN project transitions to real-world scale-up, the need for collecting data to assess effectiveness will cease, meaning that this login could be discontinued. This change will facilitate easier access to the evidence-based information provided in the NN resource for all parties involved.

Another important consideration from staff regarding future implementation efforts involves the importance of early stakeholder engagement. This was deemed crucial to ensure sufficient buy-in of NN within the counties and their municipalities, and a sense of ownership among MCHC staff. These propositions are supported by the study of Mikkelsen et al. (2016), assessing multi-level multi-component community interventions for healthy living [50]. In their study, limited involvement and support of relevant stakeholders were identified to be obstacles to intervention adoption. Additionally, building strong stakeholder networks before and during implementation was deemed to be supportive of the implementation process and essential for long-term success and sustainability [50]. In the NN project this may entail contacting all relevant MCHCs in the counties early, to engage department leaders and coordinators early. It is also worth acknowledging that staff themselves suggested that increased involvement and support from management, or assigning an accountable individual, could be beneficial for future implementation efforts. Studies have recognized supportive management and selection of staff members as champions to be important facilitators for implementation. For instance, Love et al. (2022), in a study evaluating factors contributing to the sustained implementation of the early childhood obesity prevention intervention INFANT, found management support to be one of the main enablers to sustain implementation [51]. Furthermore, the use of champions has been described to be positively associated with the use and adoption of innovations in healthcare [36, 52]. In the present study, one of the implementation strategies entailed regular contact with MCHC coordinators to provide support during the implementation process if needed. However, these coordinators were not assigned to have championing roles. Within implementation science, a champion can be described to be an individual within an organization who actively promotes and participates in leading implementation initiatives [53]. Engaging one or more individuals in championing roles and training them in how to use the NN resource, could possibly have facilitated the engagement with and use of the NN resource among MCHC staff during the first phase of the study. Additionally, identifying and preparing champions has been suggested to be among the strategies within the ERIC taxonomy to be of high importance and feasibility [54]. It could thus be argued that ensuring sufficient manager support and identifying and preparing champions are strategies necessary to help scale up and sustain early life interventions in real world contexts.

The findings from this study demonstrate that implementing e-learning resources in an MCHC setting is highly feasible. However, several measures should be considered to ensure the scalability and long-term sustainability of these resources.

For the MCHCs the following measures may be considered (1) *Early staff involvement in intervention development.* Engage staff in the early stages of intervention development to ensure it aligns with their work and is perceived as relevant and useful. (2) *Sufficient education and training.* Provide comprehensive training and educational activities focusing on the core components and practical applications of the intervention, ensuring staff feel confident and understand how to effectively utilize it within their setting. (3) *Implementation planning.* Encourage staff to develop detailed plans outlining when and how the intervention will be used, to foster staff engagement and increase the likelihood of sustained use. (4) *Early management involvement in scale up efforts.* Involve management and other key stakeholders within the MCHC early when scaling up implementation efforts to secure adequate support, integrate the intervention into the centres’ practices, and foster a sense of ownership. (5) *Identify and train champions.* Select and train key individuals who can promote and support the intervention to increase the likelihood of engagement and sustained use.

Additionally, a separate set of measures targeting parents could include (1) *Early parental involvement.* Incorporate the parental perspective early in the development process to ensure the intervention meets their needs, thereby enhancing their engagement and utilization. (2) *Lower the threshold to access intervention.* Reduce or eliminate barriers to access the intervention, such as complex questionnaires, to make it easier for parents to use. (3) *Integration and wide promotion.* Ensure the intervention is integrated and promoted across various platforms and settings familiar to parents, to enhance awareness, visibility and accessibility.

Employing these measures may enhance the adoption and utilisation of digital early life nutrition interventions such as NN, thereby facilitating their transition to real-world practice during large-scale rollouts.

### Strengths and limitations

The study has some limitations and strengths worth acknowledging. One limitation is that our findings are based on the experiences of a small and homogenous participant group, consisting of employees who work at the only MCHC within the municipality, albeit in different units. Additionally, in the present study, MCHC staff provided many reflections on the perceived parental experience, functioning as intermediaries for parents’ perspectives. Such a proxy approach limits the ability to comprehensively grasp the full spectrum of parental experiences related to implementing NN. To minimize participant burden, it was deemed unfeasible to recruit parents for qualitative interviews during the first phase of the NN study. However, including the parental perspective could have enriched the findings provided, and such evaluations should be considered as part of the scale-up efforts.

Nevertheless, a strength of this study is its narrow aim, focusing on NN implementation within the MCHC, making it possible to achieve a thorough and detailed understanding of this specific topic. Additionally, participants hold characteristics that are highly specific to the study aim, as they are the first to have had NN available at their MCHC. Their insights are thus invaluable to help determine measures needed to improve future implementation efforts. According to Malterud et al. (2016), it can therefore be argued that the present study holds high information power [55], despite the small sample size.

Lastly, a notable strength of this study is that the authors involved in the interviews (NGO, CH) and analyses (NGO, CH, FNV, ACM) are women who have been deeply involved in the NN project’s development and implementation. The authors’ involvement from the beginning of the project has given us a comprehensive understanding of the process and strategies used to implement the NN resource. This includes the specific measures that MCHC staff had to navigate throughout the implementation phase. This involvement has enabled the authors to interpret the staff’s feedback and perspectives with greater depth and insights, in turn contributing to a deep contextual understanding that informed and enriched the interpretation of the data. Additionally, through the project the interviewers were also able to establish relationships with the participants, aiding the communication during interviews and possibly strengthening the dialogue. This may have contributed to create a sense of security in which participants were able to express and share their diverse perspectives, including critical input.

## Conclusions

The findings of this study suggest that MCHC staff viewed the NN resource as well-aligned with their work priorities and recognized its potential as a useful tool for addressing diet-related topics with parents. This indicates that NN is a suitable resource for integration into routine practice within an MCHC setting. However, the study also highlights opportunities for improving engagement and utilization of the resource, both among staff and parents. To effectively implement evidence-based digital interventions like NN in real-world settings, the present study proposes several strategies that should be prioritised. Early involvement of staff in development, targeted training and clear implementation planning could be beneficial. Additionally, engaging management and identifying champions within the MCHC might support adoption. For parents, involving them early in the intervention development process, reducing access barriers, and promoting the resource across multiple platforms to improve visibility and usage could also be valuable. Incorporating these measures during implementation efforts is likely to be important for effectively integrating early life digital resources into MCHC settings, ultimately benefiting parental efforts to improve early life outcomes.

## Supplementary Information

Supplementary Material 1.

Supplementary Material 2.

## Data Availability

The dataset supporting the conclusion of this article is included within the manuscript (and the additional files). The full datasets (transcripts) used during the current study are not publicly available due to the privacy and confidentiality of our research participants but are available from the corresponding author upon reasonable request.

## Abbreviations

ECEC: Early Childhood Education and Care
NN: Nutrition Now
MCHC: Maternal and Child Healthcare Centre
ERIC: Expert Recommendations for Implementing Change
