# Supplementary material for: Implementing an early-life nutrition intervention through primary healthcare: staff perspectives
Source: BMC Health Serv Res. 2024 Sep 20;24:1106. doi: 10.1186/s12913-024-11582-z (PMC11414156; doi:10.1186/s12913-024-11582-z)
Supplement: Supplementary file 1 — Supplementary Material 1. [file 12913_2024_11582_MOESM1_ESM.docx]

**Additional file 1.** Interview guides with public health nurses, midwives, and the department leader of the Maternal and Child Healthcare Centre

| **Group interview with public health nurses** | **Group interview with midwives** | **Interview with department leader** |
| --- | --- | --- |
| 1. What has it been like to be involved in this project so far? | 1. What has it been like to be involved in this project so far? | 1. From your point of view as a manager, how have you experienced being involved in the Nutrition Now project so far? |
| 1. In your experience, how does Nutrition Now work as a resource to address infant and toddler nutrition with parents?    1. What works well?    2. Have you been in situations where Nutrition Now has not worked well?    3. Can you describe what didn't work, what was it about? | 1. In your experience, how does Nutrition Now work as a resource to address infant and toddler nutrition with pregnant people?    1. What works well?    2. Have you been in situations where Nutrition Now has not worked well?    3. Can you describe what didn't work, what was it about? | 1. What made you, as a healthcare centre, decide to join the Nutrition Now project? |
| 1. To what extent do you feel the resource is relevant to you as employees? | 1. To what extent do you feel the resource is relevant to you as employees? | 1. Was the institutional agreement between the university (UiA) and the municipality important in enabling us to implement Nutrition Now?    1. In what way? |
| 1. Are there parts of the Nutrition Now resource you have used in your work?    1. Which ones?    2. In what way have you used these in particular?    3. What is it that has made you use these in particular? | 1. Are there parts of the Nutrition Now resource you have used in your work?    1. Which ones?    2. In what way have you used these in particular?    3. What is it that has made you use these in particular? | 1. How do you experience having Nutrition Now available as a resource in your organisation in the municipality? |
| 1. Are there parts of the Nutrition Now resource you have not visited/used?    1. Which ones?    2. What is it that has prevented you from using these in particular? Any particular reasons? | 1. Are there parts of the Nutrition Now resource you have not visited/used?    1. Which ones?    2. What is it that has prevented you from using these in particular? Any particular reasons? | 1. Have you received any feedback from the staff at the healthcare centre about their experiences with Nutrition Now?    1. What kind of feedback? |
| 1. Do you have any thoughts on how Nutrition Now can be used in counselling at the health centre? | 1. Do you have any thoughts on how Nutrition Now can be used in counselling at the health centre? | 1. Have you experienced any resistance among the staff related to participation in the Nutrition Now project?    1. Can you elaborate?    2. What is the nature of the resistance? |
| 1. How did you experience that the information about Nutrition Now was received by parents during the recruitment period? | 1. How did you experience that the information about Nutrition Now was received by pregnant people during the recruitment period? | 1. Now that we are going to implement Nutrition Now at healthcare centres throughout xxx, is there anything you think will be crucial to the success of this process?    1. Level of anchoring    2. Co-operation    3. Process    4. Other (if so, what?) |
| 1. Have you received feedback from parents about their use of the Nutrition Now resource?    1. What kind of feedback? | 1. Have you received feedback from pregnant people about their use of the Nutrition Now resource?    1. What kind of feedback? | 1. To what extent do you (as a manager) think that Nutrition Now can be a resource that the healthcare centre will continue to use?    1. What do you think it will take for you to continue using Nutrition Now?       1. Link to pages       2. Cards       3. Posters       4. Follow-up meetings/gatherings internally       5. Other (If so, what?)    2. Is there anything you as a manager can do to support continued use? |
| 1. Do you find that it has been challenging for parents to use the Nutrition Now resource?    1. In what way? | 1. Do you find that it has been challenging for pregnant people to use the Nutrition Now resource?    1. In what way? | 1. Think back to the mapping session held at the town hall. Which thoughts are you left with after attending it? |
| 1. What are your thoughts on disseminating the Nutrition Now resource to parents going forward? (New phase, now that recruitment is finalised, resource will be available to all, no questionnaire)    1. Is Nutrition Now something you would recommend to parents? | 1. What are your thoughts on disseminating the Nutrition Now resource to pregnant people going forward? (New phase, now that recruitment is finalised, resource will be available to all, no questionnaire)    1. Is Nutrition Now something you would recommend to pregnant people? | 1. Is there anything else you can think of that you would like to add? |
| 1. Do you see any challenges related to the continued use of the resource in the future for parents? | 1. Do you see any challenges related to the continued use of the resource in the future for pregnant people? |  |
| 1. Is there anything that could make it easier to continue to use/communicate about the Nutrition Now resource to parents?    1. Cards    2. Posters    3. Brochures    4. Link to pages    5. Follow-up meetings/gatherings internally    6. Send out with notice    7. What do you feel you need    8. Anything else? | 1. Is there anything that could make it easier to continue to use/communicate about the Nutrition Now resource to parents? 2. Cards 3. Posters 4. Brochures 5. Link to pages 6. Follow-up meetings/gatherings internally 7. Send out with notice 8. What do you feel you need 9. Anything else? |  |
| 1. Who has been easiest to ask to join Nutrition Now?   Who has been difficult to ask to join Nutrition Now? | 1. Who has been easiest to ask to join Nutrition Now?   Who has been difficult to ask to join Nutrition Now? |  |
| 1. Have you experienced any challenges to the Nutrition Now project as a whole?    1. Which ones?    2. Can you describe what has been the challenge?    3. How were the challenges handled? | 1. Have you experienced any challenges to the Nutrition Now project as a whole?    1. Which ones?    2. Can you describe what has been the challenge?    3. How were the challenges handled? |  |
| 1. A couple of meetings and workshops were held before the start-up of Nutrition Now. What impact has this had on the implementation of the project?    1. In what way?   PHNs that had not been part of meetings were asked:   1. Before you started Nutrition Now at the healthcare centre, a start-up meeting was held where everyone was informed about Matnyttig. What impact has this had on the implementation of the project?    1. In what way? | 1. A couple of meetings and workshops were held before the start-up of Nutrition Now. What impact has this had on the implementation of the project?    1. In what way? |  |
| 1. Health centres in xxx are now going to pass on Nutrition Now to parents, what are your thoughts on how best to anchor Nutrition Now at the individual healthcare centres? | 1. Health centres in xxx are now going to pass on Nutrition Now to parents, what are your thoughts on how best to anchor Nutrition Now at the individual healthcare centres? |  |
| 1. In one of the last meetings before start-up, you were given brochures, posters, and cards for recruitment. How important was it that you received the material physically rather than printing it out yourself?    1. Do you want small cards with a QR code just to access the Nutrition Now resource?    2. Do you still want posters? | 1. In one of the last meetings before start-up, you were given brochures, posters, and cards for recruitment. How important was it that you received the material physically rather than printing it out yourself?    1. Do you want small cards with a QR code just to access the Nutrition Now resource?    2. Do you still want posters? |  |
| 1. Do you have any thoughts or experiences regarding the Nutrition Now resource or its implementation that we haven't discussed? | 1. Do you have any thoughts or experiences regarding the Nutrition Now resource or its implementation that we haven't discussed? |  |
