# Supplementary material for: Implementing an early-life nutrition intervention through primary healthcare: staff perspectives
Source: BMC Health Serv Res. 2024 Sep 20;24:1106. doi: 10.1186/s12913-024-11582-z (PMC11414156; doi:10.1186/s12913-024-11582-z)
Supplement: Supplementary file 2 — Supplementary Material 2. [file 12913_2024_11582_MOESM2_ESM.docx]

**Additional file 2.** Themes and subthemes generated through thematic analysis

| Themes | Subthemes | Sample quotes |
| --- | --- | --- |
| **Important resource but not always utilized** | The resource aligns with the existing practice and priorities | *“Food and sleep are the things we’re asked about or talk about the most. Along with interaction and things like that.”* (Speaker 12)  *“[…] and then there’s nutrition and diet, which is something that is very relevant and that many parents have questions about, and which we think is very important.”* (Speaker 3)  *“We talk about diet a lot, or*… *I wished we talked more about it. But it’s a topic we should cover in every consultation, so to speak, but you don’t always get as much time as you’d like.”* (Speaker 6) |
|  |  | *“[…] I’ve also promoted these recipes [in the Nutrition Now resource] and so on, because I think it’s brilliant, because it is… I try to speak positively about ordinary diet, food, and nutrition, as much as possible”* (Speaker 3)  *“[…] of different projects Nutrition Now has been of one of the things that has been really nice to present, […] for the conversations we have had, it supports the dietary advice that we will give anyway”* (Speaker 8) |
|  | Perceived as a reliable supporting tool | *“It is a place that we can inform about and in which we can be confident that there is good and correct information [about nutrition] without too much advertising and opinions”* (Speaker 3).  *“Then it’s very good that we as an institution, which are supposed to be quality assured […] can recommend it [Nutrition now]. It’s very safe, I think […]”* (Speaker 1)  *“It’s an easy thing to inform people about. It shouldn’t take many minutes if you’ve handed out the cards and… explained what it entails. If there are a lot of projects we have to inform about, between people who want to use our service, it’s a lot of stress. But it hasn’t been very demanding, the Nutrition Now [project]”* (Speaker 11)  *“I’ve thought that it’s positive that we have something more than what we have. Because in a way, we’ve only had dietary advice from the health authorities or the Directorate of Health. […] it’s nice to have something that everyone can get. Something that is easy to give out [...]”* (Speaker 6) |
|  |  | *“Nutrition Now is underpinning what we otherwise talk about […] so I feel it has been a tool for us to discuss food and lifestyle in a good way”* (Speaker 8)  *“Nutrition Now in a way highlights our emphasis on nutrition and how important it is”* (Speaker 4) |
|  | Lack of personal familiarity with the resource | *“I’ve only been on the [Nutrition Now] research page and watched the little video clip and that was all right, but I haven’t logged in. I probably could have done that, but I’ve forgotten”* (Speaker 6)  *“When I was informing, I recognized that I hadn’t logged in myself. I should have done that. […] When they [parents] then have asked questions, I haven’t really been able to answer”* (Speaker 5)  *“I’ve probably been lacking familiarity to that site [Nutrition Now resource]. It becomes hard to promote when you don’t know it that well”* (Speaker 7)    *“No, I've been in there looking [in the resource], but I haven't used it and actually retrieved information myself for when I'm counselling, so to speak. But we had to get connected, so it was a bit... It was a bit cumbersome”* (Speaker 3)  *“I think I've only barely been inside [the resource]. But it’s like a tad concerning then, when it is so difficult for me to get in…”* (Speaker 7) |
|  |  | *“I don’t believe so […]. We have in a way been through… been part of and gotten a picture of…We have not been sitting around recommending something and having no idea of what it is”* (Speaker 2) |
|  |  | *“We have a group of very experienced nurses who feel we know the basics of nutrition counselling. So, we may not have had a great need for it”* (Speaker 1)  *“But if I think about it a bit, because we do provide regular counselling on food and meals and nutrition. And that they [parents], or not to brag about ourselves, but that they feel they get what they need here.”* (Speaker 2) |
| **Parents are interested but had issues with access** | Parents seemed interested but many did not log in | *“They [parents] think it’s exciting. […] I felt it was well received when we informed them about it [Nutrition Now]”* (Speaker 9)  *“There have been many who have been interested […] [they] think it has been exciting and are positive”* (Speaker 5)  *“I believe some found it okay, while many felt that perhaps it wasn’t what they had… They might have hoped for or expected something very exciting”* (Speaker 3) |
|  |  | *“I’ve received many of the parents who have children in the ECEC facilities again. They are very positive. They say “Yes, we are familiar with that [Nutrition Now], because we have that in ECEC facility, and it’s very good””* (Speaker 9)  *“I can only reinforce what you say, those who know it (Nutrition Now) from the ECEC facilities, they wake up a little and recognise it”* (Speaker 8)  *“… they’ve spoken very favourably about the ECEC facilities. They have been very positive about what has happened in the ECEC facilities and noticed that the children are eating more varied and enjoying things they didn’t like before”* (Speaker 5) |
|  |  | *“The people who get most involved are the ones who bring it up themselves. That they find food difficult or that it’s something related to weight. So it’s always lovely with those who are interested in the topic in the first place”* (Speaker 11) |
|  |  | *“I’ve asked a number of people if they’ve logged in and had a look. Some have, and many think it’s good. And then there are some who say “Oh, no, I’ve forgotten”. So, it varies”* (Speaker 11) |
|  |  | *“[…] I think it is very difficult to ascertain whether they’ve taken any note of it”* (Speaker 8)  *“We do provide an extremely large amount of information. There are a lot of things we have to inform parents about, so the question is what they take in and what they don’t, you just don’t know”* (Speaker 9)  *“But it’s probably as you say that we talk about a lot, and then the question is how much is used when push comes to shove. That’s another thing”* (Speaker 3)  *“It’s a real eye-opener. They’re so positive when they’re here, and then they don’t do anything more about it”* (Speaker 1) |
|  |  | *“[…] and I also think they like to talk about it [diet]. […] I have the impression that they feel that it’s very personalized to their child, rather than going on app or, yeah…”* (Speaker 4)  *“I think it is important that we don’t just hand out the card [with access to the resource] and then we’ve addressed this with diet. We need to address it individually and talk about it”* (Speaker 6) |
|  | Accessing the resource presented challenges | *“There was so much to do in order to sign up, and that was quite a big obstacle for many”* (Speaker 1)  *“It was difficult and cumbersome to log in, and the thing with the questions that were a little too intimate”* (Speaker 10)  *“No one has said anything negative. Other than login problems for a period there. […] That they couldn’t log in and that it was a bit difficult then”* (Speaker 6)  *“It is kind of tricky to get into it [the resource]. It has to be simple. Everything you are going to use has to be simple”* (Speaker 9). |
|  |  | *“It was the questionnaire that they felt was… quite a lot and took a lot of time. There were also some who, at times, had problems getting in, so they struggled with that”* (Speaker 2) |
|  |  | *“[…] because you [the other colleagues] got feedback that there was a lot to fill in, and someone else got feedback that it didn’t work at first. That was very demotivating for us at the time. That was a real blow”* (Speaker 1) |
|  |  | *“I’ve been feeling that a bit, that I offer it [the resource], but then I know that you [the research group] will collect something from them […]. I wish they could just go in and obtain information [from the resource] without having to do so much”* (Speaker 4) |
|  | Parents are subject to other competing factors | *“I think they find a lot on TikTok, not on Nutrition Now”* (Speaker 8)  *“But it must be easy for people to get in there [on Nutrition Now] and follow along, because, as I said, there are many others who are also on that same scene [addressing nutrition] and have a lot to offer. And who are also quite strong, in relation to parents.”* (Speaker 3)  *“[…] that they realize that it [Nutrition Now] is something different perhaps. Like “Why should I choose this over the platforms?” That’s a challenge. Because there’s insanely much to choose from”* (Speaker 1) |
|  |  | *“When we talk about it being hectic, it's hectic for these parents of young children. And when that phase, when breastfeeding [...] changes to solid food, that's when they start working, the mums. And they're very quick with digital solutions, but I think things that are on an app or that are quick to access, I think that would be a smart solution.”* (Speaker 2)  *“I think it's a bit like [...] that parents they [...] that they would really like to. Find it really exciting. But then there's getting the time to sit down. I think that's kind of the key”* (Speaker 7) |
| **Staff and stakeholder buy-in and commitment needed from the start** | Informational material was well received | *“About these cards […] it’s been really good to be able to give parents that too, because when we provide so much information, it’s clear that a lot of it disappears. So, it’s been really OK to have cards. So it’s been really good to have that, and also these stickers, it was perhaps a reminder for them [the parents]”* (Speaker 10)  *“I’ve been happy with those [the cards]. Because we don’t have many resources in Arabic and other languages. So, it’s been quite nice. A little more in-depth”* (Speaker 7) |
|  |  | *“It was good to have that little sticker. Because then you’ve seen on the plastic folder [weighing card provided to all parents visiting the MCHC] that you’ve handed out [the information]. So that was smart when it arrived after a while”* (Speaker 6)  *“It has been easier to talk about diet after getting those [cards] […]. It reminded me to hand them out and […] it created like a little pocket there to talk about it”* (Speaker 7) |
|  | Multilevel buy-in processes are needed | *“[..] That preliminary work […] makes it more clear for us and perhaps even easier to offer. To see how good the project is. So I think it strengthens me in wanting to pass on the offer [to parents]”* (Speaker 4)  *“No, I guess it’s been essential [workshops and meetings], in order to have, well, a basis for communicating this [Nutrition Now].”* (Speaker 2) |
|  |  | *“You have to have it all the way up to top management. That it becomes anchored in a way... that this is something the municipality should go for. If you want to create change, it may have to involve the entire structure”* (Speaker 8). |
|  |  | *“It’s an important point to talk to the managers, and maybe the coordinators as well, to get in. […] the managers must understand what this is about at least.”* (Speaker 1) |
|  |  | *“[…] management is certainly important. However, I think it’s very important that the managers don’t come and say “now you’re going to start using Nutrition Now”. Because that can be a bit annoying if you get it showed down your throat”* (Speaker 10) |
|  |  | *“People need to have a sense of ownership. That’s the most important thing. If you’re going to implement it, the employees have to think that “yes, this is us and this will help us in our work”. And that means having to meet up, or I think we need to talk to each other, to get that feeling”* (Speaker 8).  *“It’s always a little more engaging to meet people for real”* (Speaker 11) |
|  | Improvement of implementation   - Improvements in the MCHCs - Improvements for parents | *“We can perhaps have it in the procedures that we should… We often bring up food when the child is four months. And often elaborate at six months. And we have that set. But maybe we could have some fixed procedures where we talk about Nutrition Now again. Say that you can find nice recipes there. Recipes on mashed vegetables… Something like that”* (Speaker 6).  *“We have a four-month group […] and talk about transitions to solid food. So it’s a time that is…that all first-time mothers, at least, need counselling. So it could be an idea that we sort of established that we should at least show it there […] that we had that in our procedures”* (Speaker 1). |
|  |  | *“It’s about being confident and knowing the tool you’re using. So, if I had become familiar with the Nutrition Now site, I could easily be like “here you have this and…”. But that requires an effort from us then”* (Speaker 7).  *“Spend some time on it. If you’re going to embrace it and actually, well, get a bit inspired, you have to spend some time on it”* (Speaker 5)  *“Maybe we should have had a seminar day together. Where we kind of sat together and went into Nutrition Now […]. Spent some time and clicked. Gotten it under our skin a bit…the material”* (Speaker 7)  *“That we get an information meeting from you first. And then familiarize ourselves with the content. […] Maybe get some suggestions on how we can use it. But also that each healthcare centre figures out how to address it, and at what time”* (Speaker 6). |
|  |  | *“The management must remind people that this exists. That there are brochures, and that posters are put up, and that materials are easily accessible to everyone. That it becomes something we talk about”* (Speaker 11)  *“[…] that someone holds the lines a bit and check in, like “how are you doing now”* (Speaker 6). |
|  |  | *“I think that… The majority [of MCHCs] probably have some form of organization with a subject coordinator or someone who is responsible. I think… it’s very useful that they are central people”* (Speaker 12)  *“Maybe it’s good to have one person in charge who you can talk to. Figure out those practical things. So there won’t be any gaps or problems”* (Speaker 7)  *“It’s very smart to have one or two people in charge at the healthcare centre. […] [That] there is someone who feels a little extra responsibility”* (Speaker 6). |
|  |  | *“When it starts to become relevant, i.e. when they start to realise that the child needs a different type of food. To start talking about it when the child is six weeks, three months, that’s too early. […] also if it’s too late, because by then they’ve kind of gotten into […] a pattern”* (Speaker 3).  *“It has often worked very well to inform at two years of age, precisely because the children are very picky and frustrated their parents for not eating. I’ve sometimes given it very early. And then you see… They’re not… In the first few months they’re not very interested, or that’s not where they spend their time”* (Speaker 5). |
|  |  | *“You realise that the years go by, and things quickly become outdated. It may be relevant to look at the resource to see if we need to update it a little. So that it always seems new and updated for parents who are going to use it. I think that’s very important […] to make it interesting for the population”* (Speaker 12).  *“But I just assume that if there are changes in the new guidelines and more and more is coming in terms of research and ultra-processed food, that it [the resource] will change”* (Speaker 4)  *“If it’s also updated in relation to new guidelines that will come along, then it’s even more relevant to use it [the resource], I think, because then it becomes even more important to talk to the parents and show them properly”* (Speaker 3) |
|  |  | *“There are also similar apps that parents use. And they’re very happy about that. That they can find a lot of information one place. We’ve recommended that Nutrition Now, eventually, once it’s no longer a research project… That it might be a good idea to collaborate with these overarching apps”* (Speaker 12).  *“[...] everything we really should and must talk about, they can read more about that [NØRS, app]. So, I think that more of these kinds of fluff things [...] aren't okay, I think the more you can gather on one platform, the better it is”* (Speaker 9).  *“I was thinking about that NØRS app. I mean, if it could be located there”* (Speaker 7) |

Abbreviations: ECEC, Early Childhood Education and Care.
